# The prognostic role of early tumor shrinkage in patients with hepatocellular carcinoma undergoing immunotherapy

**DOI:** 10.1186/s40644-022-00487-x

**Published:** 2022-09-24

**Authors:** Lukas Müller, Simon Johannes Gairing, Roman Kloeckner, Friedrich Foerster, Eva Maria Schleicher, Arndt Weinmann, Jens Mittler, Fabian Stoehr, Moritz Christian Halfmann, Christoph Düber, Peter Robert Galle, Felix Hahn

**Affiliations:** 1 Department of Diagnostic and Interventional Radiology, University Medical Center of the Johannes Gutenberg University Mainz, Mainz, Germany; 2 Department of Internal Medicine I, University Medical Center of the Johannes Gutenberg University Mainz, Mainz, Germany; 3 Department of General, Visceral and Transplant Surgery, University Medical Center of the Johannes Gutenberg University Mainz, Mainz, Germany

**Keywords:** Carcinoma, Hepatocellular, Immunotherapy, Response evaluation criteria in solid tumors, Diagnostic imaging, Treatment outcome

## Abstract

**Background:**

Early tumor shrinkage (ETS) has been identified as a promising imaging biomarker for patients undergoing immunotherapy for several cancer entities. This study aimed to validate the potential of ETS as an imaging biomarker for patients undergoing immunotherapy for hepatocellular carcinoma (HCC).

**Methods:**

We screened all patients with HCC that received immunotherapy as the first or subsequent line of treatment at our tertiary care center between 2016 and 2021. ETS was defined as the reduction in the sum of the sizes of target lesions, between the initial imaging and the first follow-up. The ETS was compared to the radiologic response, according to the modified response evaluation *criteria* in solid tumors (mRECIST). Furthermore, we evaluated the influence of ETS on overall survival (OS), progression-free survival (PFS), and the alpha-fetoprotein (AFP) response.

**Results:**

The final analysis included 39 patients with available cross-sectional imaging acquired at the initiation of immunotherapy (baseline) and after 8–14 weeks. The median ETS was 5.4%. ETS was significantly correlated with the response according to mRECIST and with the AFP response. Patients with an ETS ≥10% had significantly longer survival times after the first follow-up, compared to patients with < 10% ETS (15.1 months vs. 4.0 months, *p* = 0.008). Additionally, patients with both an ETS ≥10% and disease control, according to mRECIST, also had significantly prolonged PFS times after the initial follow-up (23.6 months vs. 2.4 months, *p* < 0.001).

**Conclusion:**

ETS was strongly associated with survival outcomes in patients with HCC undergoing immunotherapy. Thus, ETS is a readily assessable imaging biomarker that showed potential for facilitating a timely identification of patients with HCC that might benefit from immunotherapy.

**Supplementary Information:**

The online version contains supplementary material available at 10.1186/s40644-022-00487-x.

## Background

Hepatocellular carcinoma (HCC) is the most common primary liver cancer and one of the worldwide leading causes of cancer-related deaths [1]. Systemic therapy is the mainstay treatment for patients in advanced tumor stages and for patients that experienced a failure with previous surgical or locoregional treatments [2]. In addition to the previously recommended tyrosine-kinase inhibitors, sorafenib and lenvatinib, immunotherapeutic agents have gained importance over recent years [3, 4]. Initially applied mainly for patients after tumor progression in first or second line of systemic treatment, the results of the IMbrave150 trial led to a paradigm change and the combination of the checkpoint inhibitor atezolizumab and the VEGF antibody bevacizumab is now the first line treatment for patients with advanced HCC [5–7]. Furthermore, ongoing trials are investigating the potential of several other immunotherapeutic agents, both for the treatment of advanced HCC and for the potential treatment of earlier-stage tumors [4, 8, 9].

Despite the promising prognostic results of immunotherapy for most patients with advanced HCC, one third of patients with advanced HCC did not benefit from immunotherapy, and up to one fourth developed high-grade immune-related adverse events [10]. Thus, not all patients benefit equally. In clinical reality, one of the greatest challenges is the identification of patients most likely to benefit from immunotherapy. To date, we lack biomarkers that can predict the response to immunotherapy, and thus, provide guidance in clinical decision-making. To facilitate decision-making, novel biomarkers are needed, particularly as the treatment with those new agents distinctly differs from the treatment with tyrosine kinase inhibitors [9, 10]. Apart from tumor cell-derived and tumor microenvironment-derived biomarkers, in advanced HCC stages, specific laboratory parameters, like alpha-fetoprotein (AFP), and its changes during treatment, and inflammatory parameters, like C-reactive protein, have shown strong correlations with patient prognosis [10–12]. However, little is known about the potential of imaging biomarkers for predicting long-term outcomes and for identifying patients that might benefit from immunotherapy.

Early tumor shrinkage (ETS) could function as one of those novel imaging biomarkers: ETS is defined as the relative reduction in tumor size between the baseline treatment and the first follow-up investigation. Originally identified as an imaging biomarker for patients with colorectal cancer undergoing chemotherapy, ETS was also shown to be predictive of outcomes in patients with other cancer entities [13–16]. Several recent studies identified ETS as a highly promising imaging biomarker, specifically for outcomes in patients treated with immunotherapy [17–19]. Moreover, ETS was identified as a superior parameter for assessing the treatment response, compared to the conventional response evaluation *criteria* in solid tumors (RECIST) and the modified RECIST (mRECIST) criteria. However, studies are scarce on ETS in patients with HCC. To date, ETS has only been investigated for HCC treatments with tyrosine-kinase inhibitors [20, 21]. Thus, the role of ETS remains unclear for patients with HCC that are treated with immunotherapeutic agents.

The ultimate goal when applying response criteria is to identify patients likely to benefit from continuation of the current treatment or patients more likely to benefit from alternative treatments, all in the light of an improved overall survival outcome. Both conventional and modified RECIST criteria establish a reduction in the maximum tumor diameter by 30% as partial response, however, new targeted therapies may be effective without showing such a decrease in imaging [22]. Although mRECIST has outperformed RECIST in prognosis prediction, correlation with the overall outcome varied tremendously in previous studies on patients with HCC and systemic treatment [21]. Thus, the cut-offs and criteria might be only partially suitable for these patients. Furthermore, the assessment of the aforementioned criteria is complex. Therefore, an additional and easy-applicable scoring system like ETS might help to complement conventional response criteria and therefore further improve patient selection and treatment decision-making.

Based on the promising preliminary work, our study hypothesis was that ETS is a highly potential and prognostic imaging biomarker for patients with HCC and immunotherapy.

## Methods

The Ethics Committee of the Medical Association of Rhineland Palatinate, Mainz, Germany approved this study (permit number 837.199.10). The requirement for informed consent was waived, due to the retrospective nature of the study. This report followed the guidelines for transparent reporting of a multivariable prediction model for individual prognosis or diagnosis (TRIPOD )[23].

### Patients

**Fig. 1:**
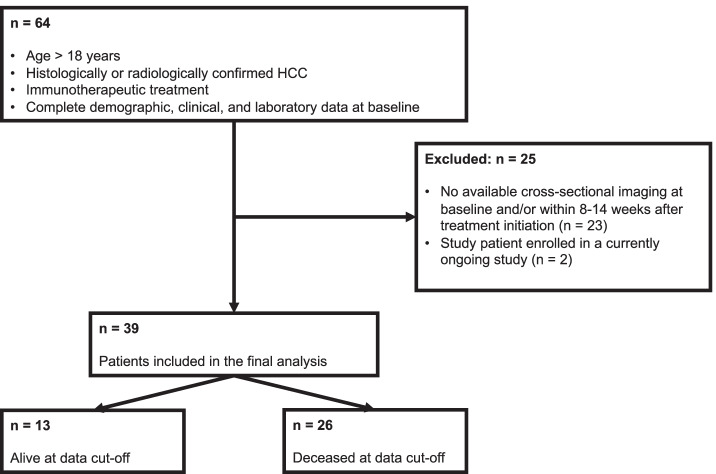
Flowchart of the patient selection process for this study. HCC: hepatocellular cancer

This retrospective study included all patients with HCC that presented in our dedicated HCC outpatient clinic between May 2016 and August 2021 for immunotherapy initiation. Inclusion criteria were: (1) age > 18 years, (2) histologically or image-derived HCC diagnosis based on the European Association for the Study of the Liver (EASL) criteria, (3) scheduled to receive immunotherapy treatment, (4) available cross-sectional imaging acquired at immunotherapy initiation (baseline) and at 8–14 weeks after the initiation of immunotherapy (short-term follow-up), (5) adequate image quality, (6) complete demographic, clinical, and laboratory data at baseline and during follow-up. Of the 64 treated patients, 39 (60.9%) fulfilled all the inclusion criteria (Fig. 1).

### Diagnosis, treatment, and follow-up

As previously reported, HCC was diagnosed, based on histological or image-derived EASL criteria [2, 24]. The decision to initiate immunotherapy was determined through discussion by an interdisciplinary tumor board prior to treatment, which consisted of hepatologists/oncologists, diagnostic and interventional radiologists, visceral surgeons, pathologists, and radiation therapists. All patients received contrast-enhanced multiphasic computed tomography (*n* = 35) or magnetic resonance imaging (*n* = 4) prior to treatment initiation and during follow-up. Imaging modality was the same at baseline and during follow-up for all patients. Follow-up consisted of a clinical examination, blood sampling, and cross-sectional imaging. Follow-ups were typically repeated every 6 to 12 weeks.

### Assessments of radiologic response, ETS, and AFP response

Radiologic responses were assessed with mRECIST criteria [2, 25]. ETS was defined as the relative change of the sum of the largest diameters of all target lesions, between treatment baseline and the first follow-up. Definition of the target lesions was based on the definitions of the mRECIST criteria, which have shown a high grade of reproducibility in previous studies [2, 25–28]. Images were analyzed by a board-certified consultant radiologist with more than 10 years of experience in liver imaging. Thus, we followed the recommendation that experienced and specifically trained radiologists are important to minimize variability in the evaluation of response [29]. The reader had information on the clinical history of the patient and the initiated immunotherapy but was blinded for the clinical outcome of the patients to simulate a situation radiologists face in their routine evaluation of these patients. Supplementary Fig. 1 shows an example of an ETS assessment. Furthermore, Supplementary Figs. 2 and 3 show patient examples. Moreover, disease control (defined as complete response (CR), partial response (PR) or stable disease (SD)) was used to dichotomize patients according to their mRECIST response as previously reported [30]. The AFP response was calculated as the percentage change in AFP, between baseline and the first follow-up, for all patients with an initial AFP ≥20 mg/dl, as previously reported [31]. An AFP increase ≥20% was classified as progression, and any AFP decrease was classified as a therapeutic response [31].

### Statistics

Statistical analyses and graphic design were performed in R 4.0.3 (A Language and Environment for Statistical Computing, R Foundation for Statistical Computing, http://www.R-project.org; last accessed 15 Mar 2022). Continuous data are reported as the median and interquartile range (IQR). Categorical and binary baseline parameters are reported as absolute numbers and percentages. Categorical parameters were compared with Fisher’s exact test. Continuous parameters were compared with the Mann-Whitney test. Survival analyses and Kaplan-Meier analyses were performed with the packages “survminer” and “survival” (https://cran.r-project.org/package=survminer, https://CRAN.R-project.org/package=survival, accessed 15 Mar 2022). The optimal ETS cut-off value with regard to survival stratification was determined using the R package survminer (last accessed 15 March 2022) and compared to the previously published cut-offs of 10 and 20% [20, 21]. We calculated OS and PFS starting from the first follow-up imaging procedure, because this was the first time that the ETS and the response according to mRECIST could be assessed. Log-rank tests were performed to compare survival times. Cox proportional hazards regression models were used to assess hazard ratios (HRs), and the corresponding 95% confidence intervals (CIs) were used to determine the effect of risk stratification. *P*-values < 0.05 were considered significant.

## Results

### Study population

A total of 39 patients were included in the final analysis. Of the included patients, 31 (79.5%) were male and the median age at baseline was 67 years (IQR: 62–74 years). The median time between baseline and follow-up imaging procedures was 78 days (range: 64–90 days). Detailed baseline characteristics are shown in Table 1.

**Table 1:** Baseline characteristics of the included patients

Parameter	All patients (*n* = 39)
Age, years, median (IQR)	67 (62–74)
Sex, n (%)	
Female	31 (79.5)
Male	8 (20.5)
Etiology of cirrhosis, n (%)	
Alcohol	13 (33.3)
Viral	9 (23.1)
Other	9 (23.1)
No cirrhosis	8 (20.5)
Child-Pugh stage, n (%)	
A	20 (51.3)
B	9 (23.1)
C	2 (5.1)
No cirrhosis	8 (20.5)
ECOG, n (%)	
≤1	33 (84.6)
> 1	6 (15.4)
BCLC stage, n (%)	
B	3 (7.7)
C	34 (87.2)
D	2 (5.1)
Portal vein invasion, n (%)	
Yes	20 (51.3)
No	19 (48.7)
Distant metastasis, n (%)	
Yes	22 (56.5)
No	17 (43.5)
Immunotherapy agent, n (%)	
Atezolizumab + Bevacizumab	22 (56.5)
Pembrolizumab	10 (25.6)
Nivolumab	7 (17.9)
Line of systemic treatment, n (%)	
First	19 (48.7)
Second	12 (30.8)
Third or higher	8 (20.5)
Previous therapy, n (%)	
Yes	32 (82.1)
No	7 (17.9)
Subsequent therapy, n (%)	
Yes	15 (38.5)
No	24 (61.5)
Number of target lesions, n (%)	
1	6 (15.4)
2	21 (53.8)
3	6 (15.4)
4	4 (10.3)
5	2 (5.1)
Sum of the sizes of all target lesions, mm, median (IQR)	61 (49–111)
AFP, ng/ml, median (IQR)	141 (15–2548)
Albumin, g/l, median (IQR)	31 (28–35)
Bilirubin, mg/dl, median (IQR)	1.2 (0.7–2.2)
INR, median (IQR)	1.2 (1.1–1.3)
Creatinine, mg/dl, median (IQR)	0.8 (0.7–1.1)

*ECOG* Eastern Cooperative Oncology Group, *BCLC* Barcelona Clinic Liver Cancer, *AFP* alpha-fetoprotein, *INR* International Normalized Ratio

### ETS and mRECIST

**Fig. 2:**
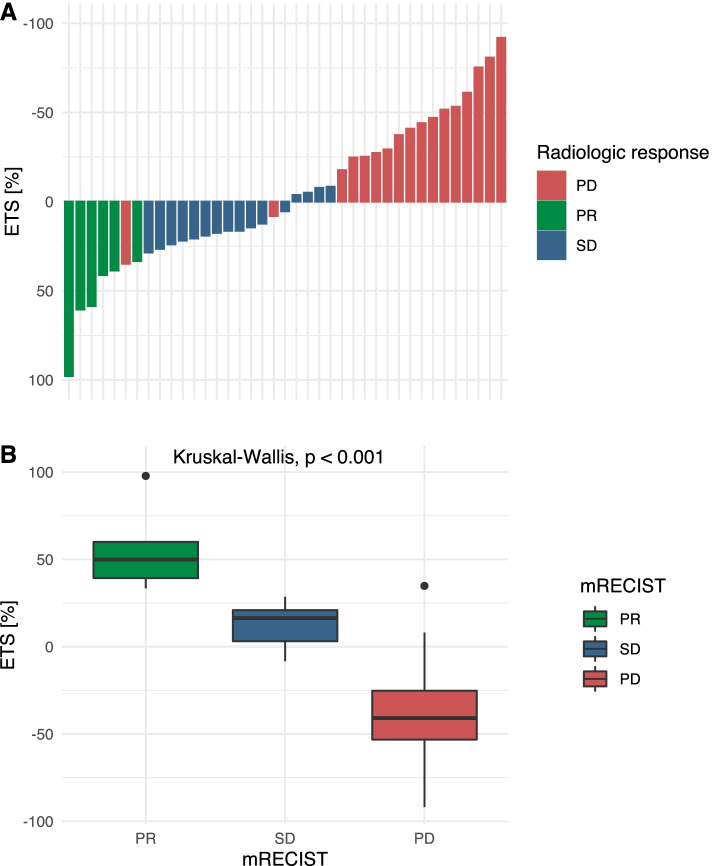
Early tumor shrinkage (ETS) distribution among patients with different responses to immunotherapy for hepatocellular carcinoma. A. Waterfall plot displays the ETS and treatment response, according to mRECIST, for each patient; B. Boxplot illustrates the ETS in each mRECIST patient group; mRECIST: modified response evaluation *criteria* in solid tumors; PR: partial response; SD: stable disease; PD: progressive disease

Overall, at the first follow-up, tumor size increased in 19 (48.7%) patients and decreased in 20 (51.3%) patients. The median ETS for the entire cohort was 5.4% (IQR: − 33.3 – 22.9%, range: − 91.8 – 97.8%). Figure 2A shows the overall distribution of ETS for all patients.

Among the 39 patients, 32 (82.1%) had undergone previous treatment (Table 1). In these patients the median ETS was 14.5% (IQR -6.33 – 27.0%). In patients without previous treatment (*n* = 7, 17.9%), the median ETS was 0.9% (IQR -41.7 – 22.4%, *p* = 0.31). For the 19 (48.7%) patients for whom immunotherapy was the first-line of systemic treatment, the median ETS was 14.5% (IQR -27.0 – 29.9%), while the median ETS of the 20 (51.3%) who received immunotherapy as second or higher line was − 6.0% (IQR -38.2 – 17.4%, *p* = 0.36).

Of the patients with PD (*n* = 17), *n* = 15 (88.2%) of the patients showed a threshold growth of their target lesions. Of those, *n* = 12/15 had new lesions detected at the first follow-up as well. Additionally, *n* = 2/17 patients showed no threshold growth of the target lesions, but had new lesions detected at the initial follow-up.

The median ETS values were 49.9% (IQR: 39.2–60.0%) for patients with PR (*n* = 6, 15.4%) and 16.4% (IQR: 3.1–21.0%) for patients with SD (*n* = 16, 41.0%). Patients with PD (*n* = 17, 43.6%) had a median increase in the tumor burden of 40.9% (IQR: − 25.2 – 53.2%; *p* < 0.001).

### Survival analysis

Optimal stratification for our own cohort was reached when using a cut-off between 8.1 to 12.3%. Thus, we adapted the previously reported cut-off of 10.0% for ETS as an appropriate value for our cohort of HCC patients undergoing immunotherapy.

**Fig. 3:**
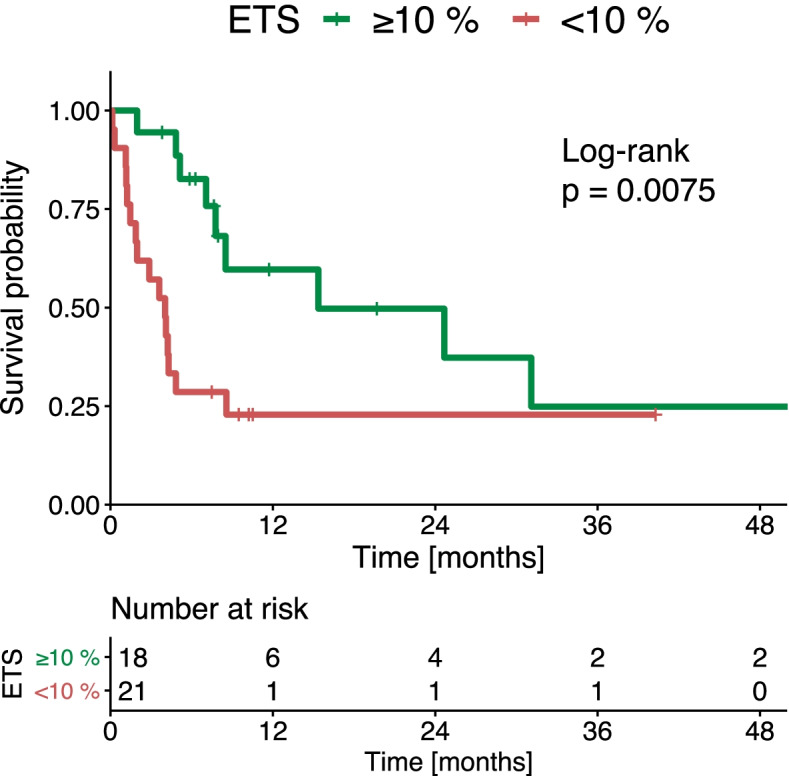
Kaplan-Meier curves show overall survival of patients treated with immunotherapy for hepatocellular cancer, stratified according to the amount of early tumor shrinkage (ETS)

A total of 18 (46.2%) patients had an ETS ≥10%. The median OS after the first follow-up study was 15.1 months, for patients with an ETS ≥10%. That OS was significantly longer than the OS in patients with an ETS < 10% (4.0 months, *p* = 0.008, Fig. 3). Using the cut-off of 20.0%, patients with an ETS ≥20.0% had a median OS of 24.3 months, while patients with an ETS < 20% had a median OS of 4.2 months (*p* = 0.082, Supplementary Fig. 4).

**Fig. 4:**
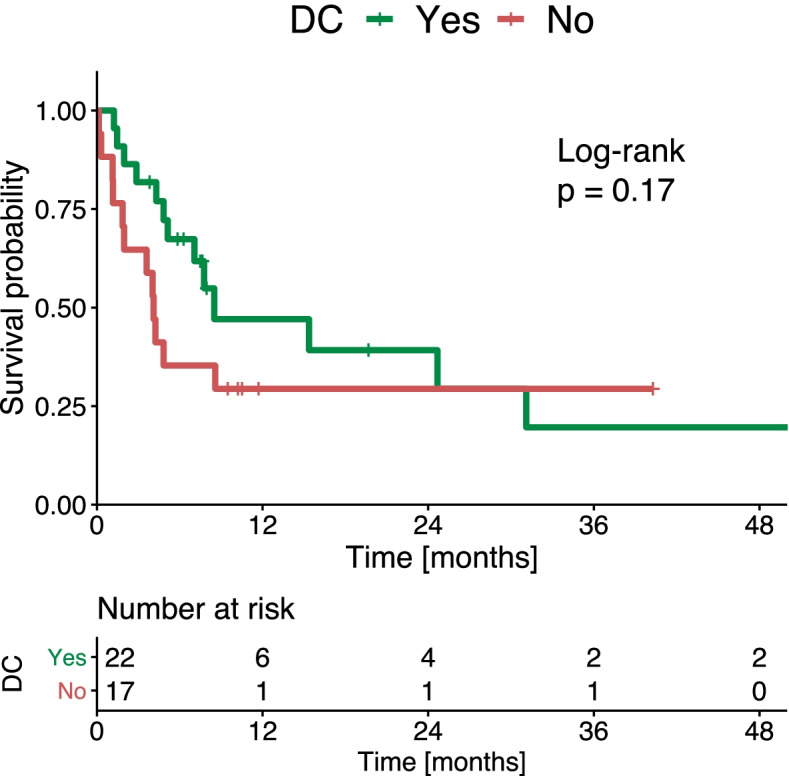
Kaplan-Meier curves show overall survival for patients treated with immunotherapy for hepatocellular cancer, stratified according to whether they achieved disease control (DC)

Overall, 22 (56.4%) patients achieved disease control, which was defined as complete response (CR), partial response (PR) or stable disease (SD) according to mRECIST. After the first follow-up, the median OS times were 11.2 months, for patients with disease control, and 6.9 months for patients without disease control (*p* = 0.170; Fig. 4). In a Cox hazard regression analysis, patients with an ETS < 10% at the first follow-up were more likely to die than patients with an ETS > 10% (HR: 3.0, standard error (SE): 0.4, 95% CI: 1.3–6.9, *p* = 0.011). In contrast, the likelihood of death among patients without disease control was not significantly different from that of patients with disease control (HR: 1.7, SE: 0.4, 95% CI: 0.8–3.8, *p* = 0.178).

### Progression-free survival

**Fig. 5:**
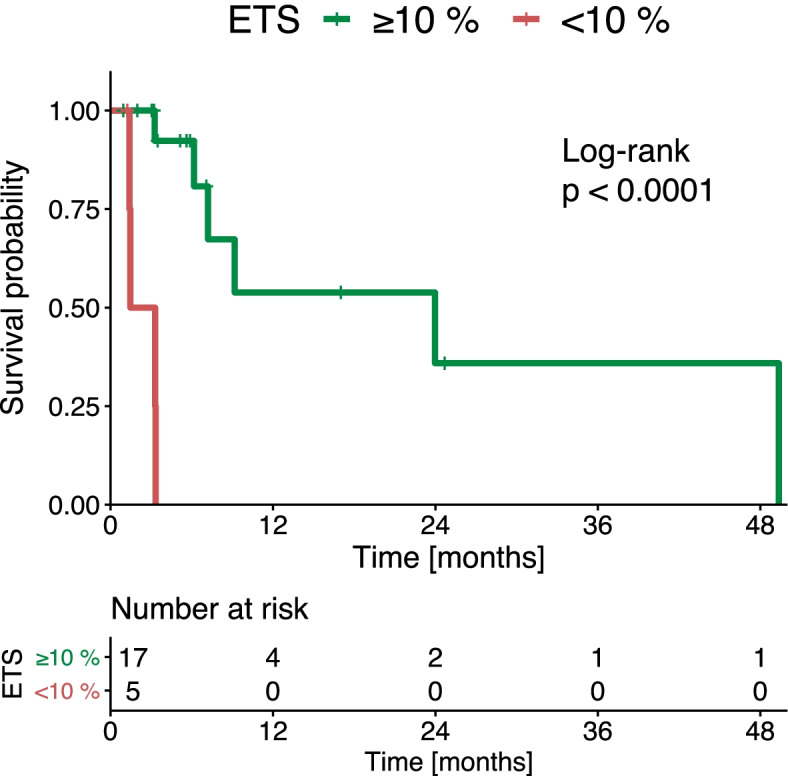
Kaplan-Meier curves show progression-free survival of patients treated with immunotherapy for hepatocellular cancer, stratified according to the amount of early tumor shrinkage (ETS)

Additionally, we analyzed the PFS for patients that achieved disease control at the first follow-up. Patients with an ETS ≥10% had a significantly higher median PFS (23.6 months) than patients with an ETS < 10% (2.4 months, *p* < 0.001; Fig. 5).

Moreover, in the subgroup of patients with disease control at first follow-up, patients with an ETS < 10% were more likely to show disease progression than patients with an ETS ≥10% (HR: 26.0, SE: 1.1, 95% CI: 2.9–241.0, *p* = 0.004).

### AFP response

**Fig. 6:**
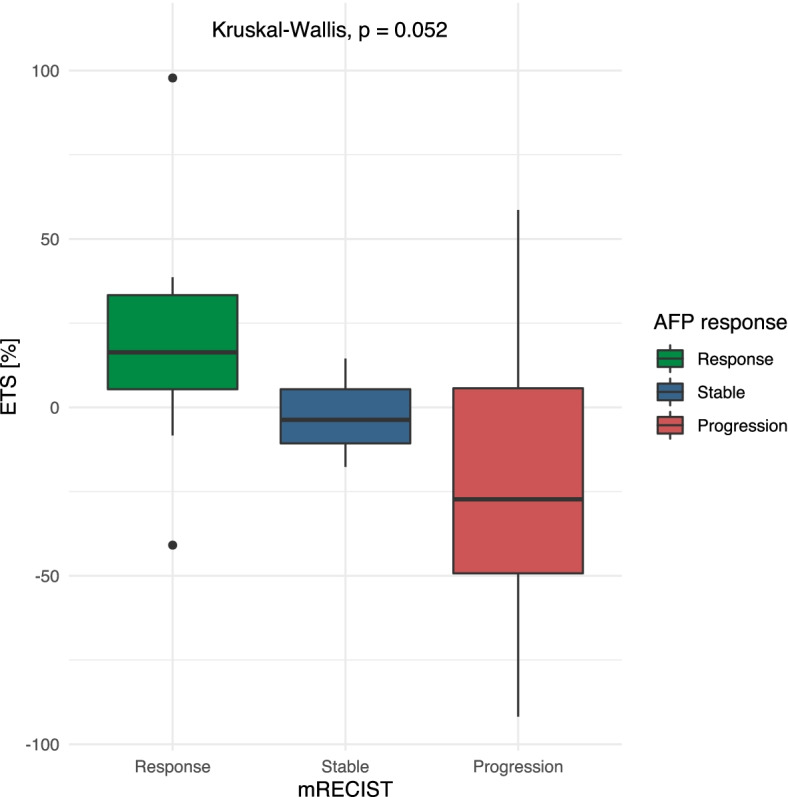
Relationship between early tumor shrinkage (ETS) and the alpha-fetoprotein (AFP) response categories, in patients treated with immunotherapy for hepatocellular cancer. mRECIST: modified response evaluation *criteria* in solid tumors

The median ETS was 16.4% (IQR: 5.4–33.3%) among patients that showed an AFP response to therapy. The median ETS values were − 3.7% (IQR: − 10.7 – 5.4%), among patients with a stable AFP, and − 27.3% (IQR: − 49.3 – 5.7%), among patients with AFP progression (*p* = 0.052; Fig. 6).

No significant differences for the laboratory parameters constituting the ALBI and MELD score (albumin, bilirubin, INR and creatinine) were observed between patients with ETS ≥10% and ETS < 10% (Supplementary Table 1).

## Discussion

In this study, we demonstrated that ETS could predict survival in patients with advanced-stage HCC undergoing immunotherapy. In addition, ETS could be used to further stratify patients with disease control at first follow-up, and it was associated with the AFP response during treatment. Thus, ETS showed high potential as an imaging biomarker for patients with HCC undergoing immunotherapy.

The IMbrave150 phase III-trial established that atezolizumab plus bevacizumab could serve as a new first-line regimen for systemic therapy-naïve patients with advanced-stage HCC. The objective response rate was 30% vs. 11% (atezolizumab + bevacizumab vs. sorafenib, based on RECIST1.1) [32]. Moreover, the HIMALAYA phase III-trial showed that the STRIDE regimen (tremelimumab/durvalumab) was superior to sorafenib, which further expanded immunotherapy options for unresectable HCC [33]. In addition, an interim analysis of the COSMIC-312 phase III trial found that the combination of atezolizumab/cabozantinib improved PFS over sorafenib, in the first-line setting [34]. As the treatment landscape broadens, biomarkers that predict the response to immunotherapy and prognosis are of pivotal importance, because they can guide clinical decision making in the context of personalized medicine.

Importantly, recent studies have indicated that not all patients benefit equally from treatment with immunotherapeutic agents [35]. Unfortunately, several candidate molecular and immunohistochemical biomarkers to identify patients likely to benefit, which are well-known from other cancer entities, showed no predictive capacity or have too low a prevalence in patients with HCC [9, 36–38]. Novel biomarkers like circulating tumor cells or composition of the gut microbiota as well as newly identified gene signatures are currently investigated and showed promising initial results in first proof-of-concept studies [10, 39, 40].

Apart from biomarkers derived from tumor tissue, peripheral blood, and feces, imaging biomarkers are under consideration for identifying patients with HCC that are most likely to benefit from immunotherapy. However, it remains unknown whether imaging biomarkers that were identified for other tumor entities might be effective for patients with HCC treated with immunotherapy. One promising imaging biomarker is ETS, which was first described as an imaging biomarker in patients with colorectal liver metastasis [13]. Results from a meta-analysis that included patients from 10 different trials indicated that ETS showed enormous potential for supporting the identification of patients that would be sensitive to treatment and patients that would benefit from treatment continuation [13]. Based on those promising results, ETS was recently identified as an imaging biomarker for various other cancer entities, and it specifically showed potential as a predictive factor in patients treated with immunotherapy [14–19].

Our results were consistent with a study that investigated ETS in patients treated with lenvatinib, by Takahashi et al., that identified a significant correlation between the AFP response and ETS [20]. Furthermore, those authors reported that ETS was highly associated with OS and PFS, calculated from treatment initiation [20]. The same results were previously observed for patients with HCC treated with sorafenib [21].

Notably, none of the laboratory parameters constituting the ALBI and MELD score did differ significantly between both ETS risk groups. However, not only the initial laboratory parameters but also their change during treatment might be relevant. For example, Granito et al. showed recently that the change of transaminases after TACE was a highly relevant predictor of treatment response [41]. One topic for future studies therefore might be the role of changes in laboratory parameters and their correlation with response and survival in patients with HCC undergoing immunotherapy.

In our study, the median ETS did not significantly differ between patients with previous treatment prior to immunotherapy and for whom the immunotherapy was the initial treatment. Furthermore, no significant difference was observed between patients who had undergone previous systemic lines of treatment and those who did not. However, our subgroups were small.

Takahashi et al. showed that ETS was highly correlated with the mRECIST criteria [20]. In their study, nearly 70% of patients with an ETS ≥10% showed an objective response, according to mRECIST. Moreover, they found that ETS showed superior predictive ability compared to the mRECIST criteria [20]. Unfortunately, the authors did not provide a subgroup analysis for the patients without progression at the initial follow-up. In the other recent study on ETS in patients with HCC treated with sorafenib, Öcal et al. suggested a cut-off of 20.0% for patient stratification [21]. In our study, patients with an ETS ≥20.0% had a prolonged survival compared to patients with an ETS < 20.0% (24.3 months vs 4.2 months). However, the optimal cut-off in our cohort was 10%, which was the same cut-off as previously published by Takahashi et al. when investigating survival of HCC under lenvatinib [20]. This suggests that under systemic treatment, a tumor reduction less than 20% or 30% already carries a significant survival advantage.

Another imaging biomarker proposed for assessing the response to treatment is the deepness of response (DpR) [13]. Similar to the ETS, the DpR is calculated based on the sum of the largest diameter of the target lesions. Originally, this parameter was identified as a potential imaging biomarker in patients with colorectal liver metastasis [13]. In a recent study, Salem et al. evaluated the DpR in patients in the IMBRAVE150 trial with HCC that received either immunotherapy (atezolizumab + bevacizumab) or tyrosine-kinase inhibition (sorafenib) [42]. In that study, patients that received immunotherapy had a higher DpR than patients that received tyrosine-kinase inhibition. The results of that study indicated that DpR showed potential as an additional, novel tool for evaluating the treatment response in patients with HCC. However, in contrast to the ETS, the DpR is defined as the maximum tumor shrinkage. Therefore, it is unclear at follow-up investigations whether maximum tumor shrinkage has yet been achieved. Thus, DpR is a retrospective measure, which is only available after progression occurs. Consequently, the DpR is not useful for assessing treatment responses at early stages, and its use is limited to clinical studies. In contrast, the ETS can be readily calculated at the first imaging evaluation; thus, it has more potential as a tool for routine clinical settings. In future studies, it could be interesting to compare the DpR with the ETS and evaluate their correlations with OS and PFS.

Since the abovementioned positive IMbrave150 results, in which the combined therapy of the immune checkpoint inhibition atezolizumab and the VEGF antibody bevacizumab showed significantly improved survival outcome, the number of trials on immune checkpoint inhibition in combination with other biological therapies for unresectable HCC has tremendously increased [5, 6, 43]. In our study, the majority of patients (*n* = 22) received the combination of atezolizumab and bevacizumab and therefore a combination of immune checkpoint inhibition and VEGF inhibition. Thus, we believe that our results are also indicative for combined treatment of immune checkpoint inhibitors and other biological therapies.

This study had several limitations. First and foremost, the study design was retrospective, and thus, it had inherent limitations. Second, the number of patients was limited. However, the dataset was well investigated as we only included patients with complete clinical, laboratory, and imaging data. Third, we included patients treated with various immunotherapeutic agents to validate the biomarker in terms of a “real-life” clinical setting. However, the small cohort meant that subgroups for each immunotherapy agent would have been too small for a well-powered analysis. Future studies should validate the ETS as an imaging biomarker for various immunotherapeutic agents and different treatment lines. Fourth, in our study, the ETS cut-off for optimal stratification ranged between 8.1 and 12.3%. This range can be attributed to the limited number of patients in our cohort. As 10.0% falls within this range, we followed this previously suggested cut-off in our study [20], which is lower than the 20.0% cut-off which has also been published [21]. Therefore, further studies investigating the influence of different ETS cut-off values for patients with HCC and immunotherapy with larger patient numbers are mandatory for optimal cut-off selection. Fifth, in our study most of the patients (*n* = 35, 89.7%) received CT imaging and only a minority of 4 patients (*n* = 4, 10.3%) received an MRI during diagnosis and follow-up. Although the current EASL guideline does not recommend one of the methods over the other, there is evidence that MRI is more sensitive in the detection of small liver lesions [2]. Furthermore, sensitivity of MRI for the detection of smaller nodules and therefore new lesions during follow-up could have been further increased with the use of liver-specific contrast agents [44].

Despite these limitations, this proof-of-concept study was the first to show a direct correlation between ETS and the survival outcomes in patients with HCC undergoing immunotherapy. Our results revealed that the ETS has huge potential as a novel imaging biomarker for patients with HCC. Moreover, the ETS may also serve as a useful addition to the conventional response categorization. Our positive preliminary results suggested that the ETS could function as an additional and easy to calculate parameter for the early identification of treatment responders. Thus, our results could serve as the foundation for further research on ETS as a novel imaging biomarker.

## Conclusion

We found that the ETS was strongly associated with survival outcomes in patients with HCC undergoing immunotherapy. Its predictive capability was further improved in the subgroup of patients with tumor control according to mRECIST. Thus, ETS is a highly promising, readily assessable imaging biomarker for patients with HCC undergoing immunotherapy. In future, ETS could identify patients that are most likely to benefit from immunotherapy, and thereby, it could support clinical decision-making.

## Supplementary Information

**Additional file 1:.** Supplementary Figure S1.

**Additional file 2:.** Supplementary Figure S2.

**Additional file 3:.** Supplementary Figure S3.

**Additional file 4:.** Supplementary Figure S4.

**Additional file 5:.** Supplementary Table 1.

## Data Availability

Data cannot be shared publicly because of institutional and national data policy restrictions since the data contain potentially identifying patient information. Data are available upon request from the Johannes Gutenberg University Mainz Medical Center (contact via radiologie-sekretariat@unimedizinmainz.de) for researchers who meet the criteria for access to confidential data (please provide the manuscript title with your enquiry).

## Abbreviations

AFP: Alpha-fetoprotein
CR: Complete response
CIs: Confidence intervals
DpR: Deepness of response
ETS: Early tumor shrinkage
EASL: European Association for the Study of the Liver
HRs: Hazard ratios
HCC: Hepatocellular carcinoma
IQR: Interquartile range
mRECIST: Modified response evaluation *criteria* in solid tumors
OS: Overall survival
PR: Partial response
PFS: Progression-free survival
PD: Progressive disease
RECIST: Response evaluation *criteria* in solid tumors
SD: Stable disease
SE: Standard error
