# Supplementary figures and images for: The prognostic role of early tumor shrinkage in patients with hepatocellular carcinoma undergoing immunotherapy
Source: Cancer Imaging. 2022 Sep 24;22:54. doi: 10.1186/s40644-022-00487-x (PMC9509639; doi:10.1186/s40644-022-00487-x)

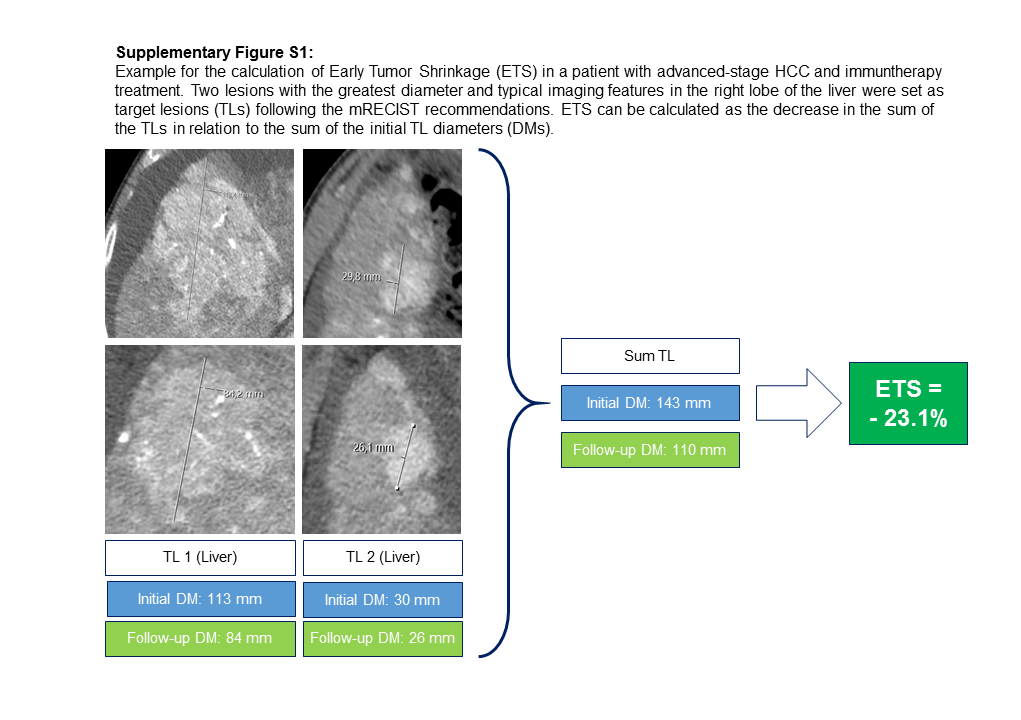

Supplement: Supplementary file 1 — Additional file 1:. Supplementary Figure S1. [file 40644_2022_487_MOESM1_ESM.tif]
