# Supplementary material for: The prognostic role of early tumor shrinkage in patients with hepatocellular carcinoma undergoing immunotherapy
Source: Cancer Imaging. 2022 Sep 24;22:54. doi: 10.1186/s40644-022-00487-x (PMC9509639; doi:10.1186/s40644-022-00487-x)

Supplementary Figure S2:
Patient example for early tumor growth after treatment initiation. In this patient who had undergone multiple previous therapies (tumor resection, tumor ablation, TACE) new liver lesions and new lung nodules were observed (TLs and non-TLs). The TL 1 in the liver had an initial diameter of 32 mm, the TLs in the lung had an initial diameter of 15 mm (TL 2) and 14 mm (TL 3). After initiation of the immunotherapy (pembrolizumab), TL 1 showed an increase in diameter to 57 mm, TL 2 to 21 mm and TL 3 to 35 mm leading to an early tumor growth of 54.0% and PD according to mRECIST.

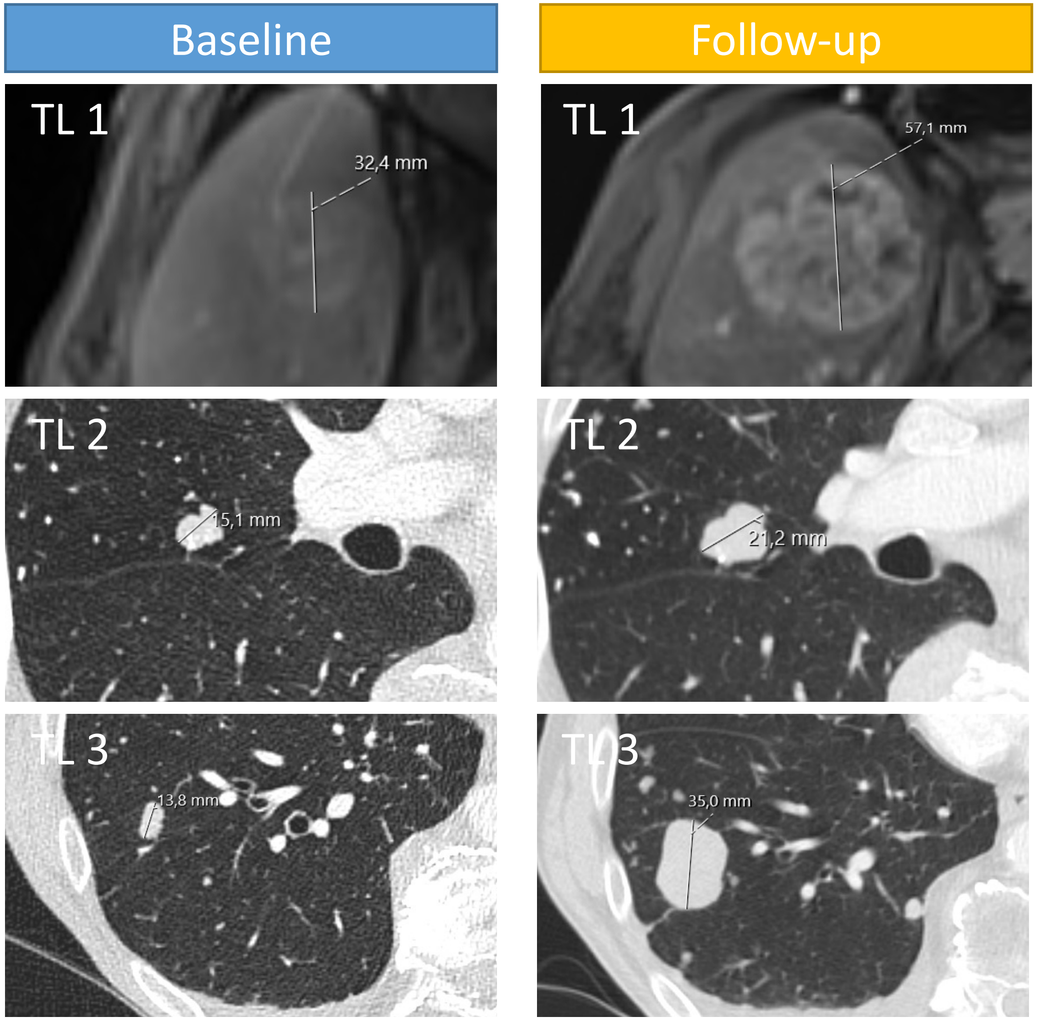

Supplement: Supplementary file 2 — Additional file 2:. Supplementary Figure S2. [file 40644_2022_487_MOESM2_ESM.pdf]
