# Supplementary material for: The prognostic role of early tumor shrinkage in patients with hepatocellular carcinoma undergoing immunotherapy
Source: Cancer Imaging. 2022 Sep 24;22:54. doi: 10.1186/s40644-022-00487-x (PMC9509639; doi:10.1186/s40644-022-00487-x)

Supplementary Figure S3:
Patient example for early tumor shrinkage.
In this patient who had undergone tumor ablation previously one abdominal lymph node metastasis (TL 1) was detected in follow-up with a diameter of 36 mm. After initiation of systemic treatment with immunotherapy (nivolumab), the lymph node metastasis shrinked to 14 mm (ETS 61.1%, PR).

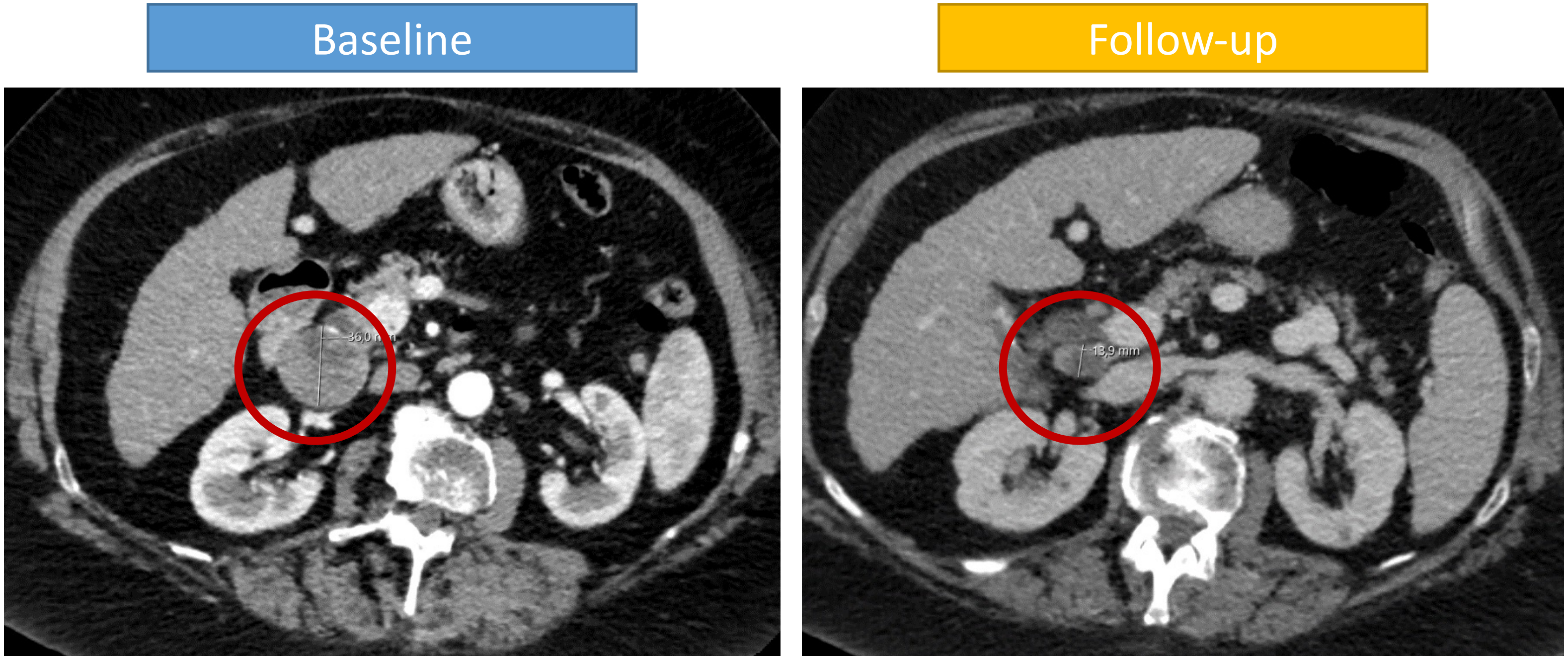

Supplement: Supplementary file 3 — Additional file 3:. Supplementary Figure S3. [file 40644_2022_487_MOESM3_ESM.pdf]
