# Supplementary material for: The prognostic role of early tumor shrinkage in patients with hepatocellular carcinoma undergoing immunotherapy
Source: Cancer Imaging. 2022 Sep 24;22:54. doi: 10.1186/s40644-022-00487-x (PMC9509639; doi:10.1186/s40644-022-00487-x)

Supplementary Figure S4:
Kaplan-Meier curves show overall survival of patients treated with immunotherapy for hepatocellular cancer, stratified according to the amount of early tumor shrinkage (ETS) with the alternative cut-off 20%

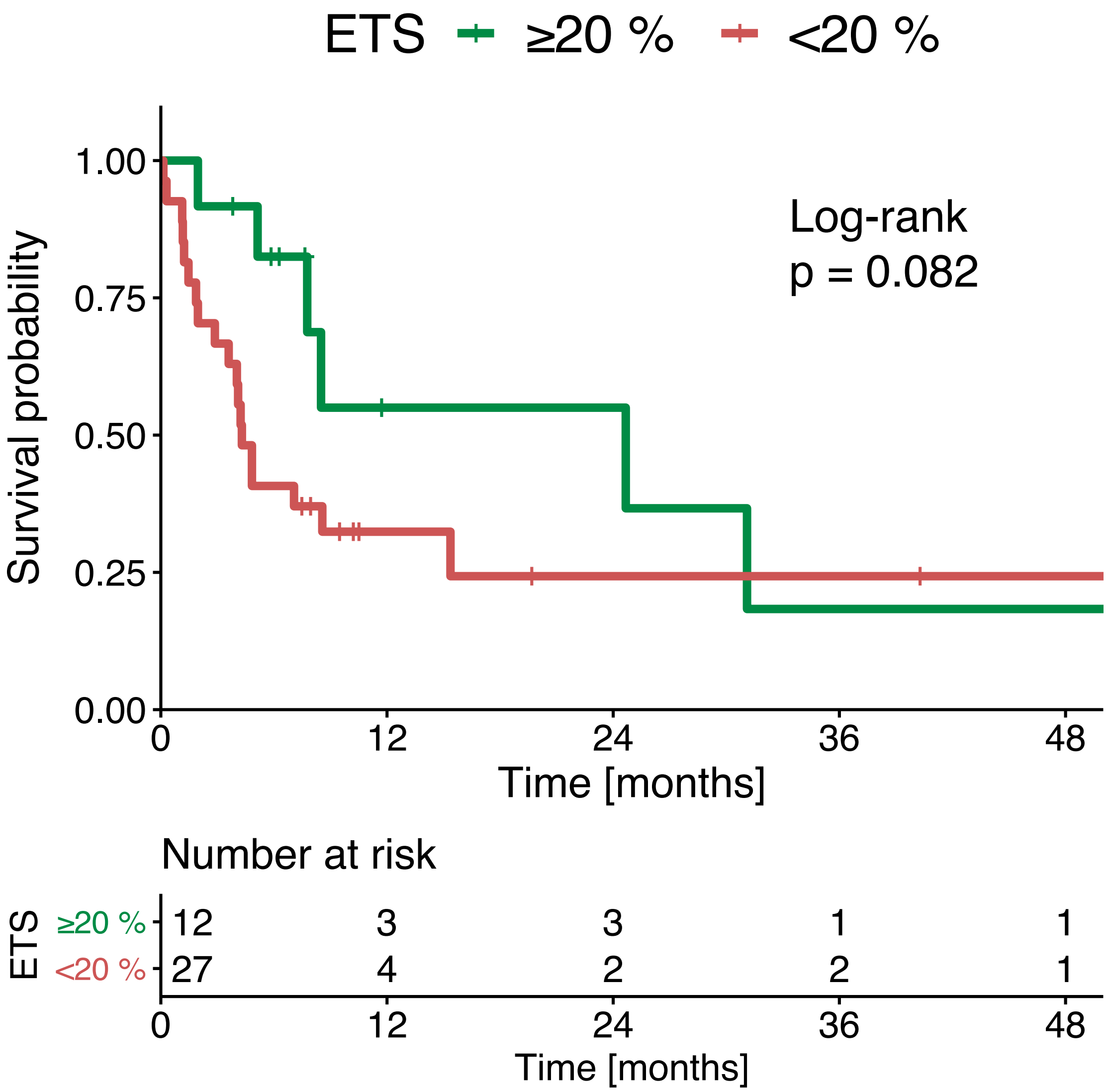

Supplement: Supplementary file 4 — Additional file 4:. Supplementary Figure S4. [file 40644_2022_487_MOESM4_ESM.pdf]
