# Supplementary material for: The prognostic role of early tumor shrinkage in patients with hepatocellular carcinoma undergoing immunotherapy
Source: Cancer Imaging. 2022 Sep 24;22:54. doi: 10.1186/s40644-022-00487-x (PMC9509639; doi:10.1186/s40644-022-00487-x)
Supplement: Supplementary file 5 — Additional file 5:. Supplementary Table 1. [file 40644_2022_487_MOESM5_ESM.docx]

**Supplementary Table 1:** Comparison of the laboratory parameters albumin, bilirubin, INR, and creatinine for both ETS risk groups

| Parameter | ETS ≥10% | ETS <10% | P-value |
| --- | --- | --- | --- |
| Albumin, g/l, median (IQR) | 32 (29 – 35) | 30 (27 – 33) | 0.454 |
| Bilirubin, mg/dl, median (IQR) | 1.2 (0.7 – 1.7) | 1.6 (0.8 – 2.5) | 0.220 |
| INR, median (IQR) | 1.3 (1.1 – 1.3) | 1.1 (1.1 – 1.4) | 0.148 |
| Creatinine, mg/dl, median (IQR) | 0.9 (0.7 – 1.1) | 0.8 (0.7 – 1.1) | 0.517 |
